# Enhancing Antimicrobial Susceptibility Testing for *Acinetobacter baumannii* Using Physiologically Relevant Culture Media and Biofilm Formation Assays

**DOI:** 10.1002/cpz1.70207

**Published:** 2025-09-22

**Authors:** Nana Yaa P. Sakyi Opoku, Arunima Mishra, Hansel Fletcher, Victor Nizet, Jacinda C. Abdul‐Mutakabbir

**Affiliations:** ^1^ Department of Basic Sciences Loma Linda University School of Medicine Loma Linda California; ^2^ Division of Host‐Microbe Systems and Therapeutics, Department of Pediatrics, School of Medicine University of California, San Diego La Jolla California; ^3^ Department of Pharmaceutical Sciences, Skaggs School of Pharmacy and Pharmaceutical Sciences University of California, San Diego La Jolla California; ^4^ Department of Clinical Pharmacy, Skaggs School of Pharmacy and Pharmaceutical Sciences University of California, San Diego La Jolla California; ^5^ Division of the Black Diaspora and African American Studies University of California, San Diego La Jolla California

**Keywords:** *Acinetobacter baumannii*, antimicrobial‐mediated resistance testing, antimicrobial susceptibility testing, biofilm formation, Mueller Hinton broth, Roswell Park Memorial Institute medium

## Abstract

*Acinetobacter baumannii* is a high‐risk pathogen associated with increased patient morbidity and mortality. Host‐pathogen interactions amplify its virulence, in part by promoting biofilm formation—a crucial factor in antimicrobial resistance and persistence. Given the bacterium's strong propensity for acquiring resistance, antimicrobial susceptibility testing (AST) is essential for guiding effective therapeutic interventions. However, discrepancies have been observed between *in vitro* AST results and therapeutic outcomes, with some antimicrobials being deemed to show *in vivo* efficacy despite appearing ineffective *in vitro*. This discordance may stem from traditional AST protocols, which rely on bacteriological media such as Mueller Hinton broth (MHB) optimized for bacterial growth but not for mimicking the host environment. Moreover, conventional AST does not account for virulence traits such as biofilm formation, which further contribute to treatment failure. Incorporating physiologically relevant culture media, such as Roswell Park Memorial Institute (RPMI) 1640 medium, alongside assessment of biofilm formation may improve the predictive value of AST. This work outlines two complementary protocols for improving AST interpretation in *A. baumannii* infections. Basic Protocol 1 compares minimum inhibitory concentration (MIC) values generated using MHB and RPMI. Basic Protocol 2 evaluates biofilm formation in MHB, tryptic soy broth (TSB; control), and RPMI, with and without antimicrobial exposure. Together, these approaches aim to inform alternative AST strategies that better reflect *in vivo* conditions and optimize therapeutic decision‐making. © 2025 The Author(s). Current Protocols published by Wiley Periodicals LLC.

**Basic Protocol 1**: Comparing *A. baumannii* minimum inhibitory concentration (MIC) results in bacteriological (MHB) versus physiological (RPMI) media

**Basic Protocol 2**: Comparing *A. baumannii* isolate(s) biofilm formation following assays completed in bacteriological culture media (MHB and control TSB) and physiological medium (RPMI)

## INTRODUCTION

*Acinetobacter baumannii*, a non‐fermenting Gram‐negative organism, is a significant cause of nosocomial infections, including ventilator‐associated pneumonia and central‐line‐associated bloodstream infections (Gidey et al., 2023; Peleg et al., 2008; Shadan et al., 2023). Because of its ability to render most therapeutics—including salvage therapies such as carbapenems and polymyxins—ineffective, the World Health Organization (WHO) has designated *A. baumannii* a global priority pathogen urgently requiring novel treatments (Abdul‐Mutakabbir et al., 2021; Asokan et al., 2019). Notably, host‐pathogen interactions contribute to its resistance (Eze et al., 2018; Pires & Parker, 2019; Sato et al., 2020). For example, during *A. baumannii* infection, host cells mount a robust inflammatory response upon recognizing pathogen‐associated molecular patterns via receptors such as TLR4 (activated by lipooligosaccharide [LOS] lipid A) and TLR9 (which detects internalized bacterial DNA). These pathways initiate signaling cascades that elevate proinflammatory cytokines and chemokines, which are essential for pathogen clearance (Chen, 2020; Li et al., 2019). In turn, *A. baumannii* employs sophisticated immune evasion strategies, including subverting the complement cascade through outer membrane proteins such as OmpA and producing a capsule that impairs opsonization and complement deposition (Shadan et al., 2023; Zhang et al., 2022).

A hallmark of *A. baumannii* pathogenicity—and a key contributor to its therapeutic recalcitrance—is biofilm formation (Gedefie et al., 2021; Harding et al., 2018). Biofilms are structured microbial communities encased in an extracellular polymeric substance (EPS) matrix, in which bacteria are shielded from antimicrobial agents and immune effectors. Within biofilms, bacteria adopt a metabolically dormant and phenotypically reversible state. Biofilms can form on both biotic and abiotic surfaces, creating highly variable microenvironments characterized by nutrient and pH gradients, which result in heterogeneous bacterial populations (Ambrosi et al., 2020; Maure et al., 2023; Roy et al., 2022). Like OmpA, biofilms modulate host‐pathogen interactions by dampening local immune responses; the matrix impedes effector cell penetration and inhibits complement activation, thereby interfering with opsonization and reducing phagocytosis. The interplay between biofilm formation and immune evasion contributes to the development of chronic infections and complicates treatment in nosocomial settings (Eze et al., 2018; García‐Patiño et al., 2017; Li et al., 2020; Magda et al., 2022; Roy et al., 2022; Schulze et al., 2021).

Treatment of *A. baumannii* infections typically relies on antimicrobial susceptibility testing (AST) to guide therapy (Pierce et al., 2023). Accurate AST is therefore paramount for enabling prompt and effective clinical decision‐making. Historically, AST has evolved through iterative improvements aimed at enhancing diagnostic precision. Early methodologies, including broth and tube dilution techniques, established the framework for quantifying the minimum inhibitory concentration (MIC) of antimicrobials (Wheat, 2001). The broth microdilution method (BMD)—an adaptation that miniaturizes tube dilution to assess multiple drug concentrations simultaneously—has since become the gold standard for AST due to its reproducibility and quantitative rigor (Khan et al., 2019).

However, although BMD provides essential MIC data, it is optimized for *in vitro* conditions and does not fully replicate the complexities of the host environment (Heithoff et al., 2023; Miller et al., 2023; Nizet, 2017). Standard AST relies on Mueller Hinton broth (MHB), a bacteriological medium optimized for bacterial growth rather than physiological relevance (Nizet, 2017; Nussbaumer‐Pröll & Zeitlinger, 2020). Studies have demonstrated that the choice of testing medium can significantly influence the observed antimicrobial susceptibility (Heithoff et al., 2023; Makris et al., 2018). In contrast, Roswell Park Memorial Institute 1640 medium (RPMI 1640, hereafter RPMI), formulated initially for propagating immortalized cell lines, more closely mimics host physiology and is increasingly used in pharmacological research. RPMI contains components such as bicarbonate, essential for maintaining physiological pH, and glutathione, a vital intracellular antioxidant that neutralizes reactive oxygen species (ROS) *in vivo*—both absent in MHB (Berti et al., 2020; Nizet, 2017). This compositional difference enhances the physiological relevance of AST, as demonstrated in studies showing potent activities of antibiotics that were evident in RPMI but absent in MHB, and corroborated by effective bacterial killing in human serum and murine infection models (Miller et al., 2023; Nizet, 2017; Rubio et al., 2024).

Additionally, standard AST primarily evaluates planktonic bacteria, overlooking biofilm formation and associated resistance mechanisms. Incorporating physiologically relevant media, such as RPMI, alongside assessments of biofilm formation and sub‐inhibitory antimicrobial effects may enhance the predictive accuracy of AST.

The objective of these protocols is to provide comprehensive guidance on evaluating the impact of growth media on AST outcomes, with the ultimately aim of identifying alternative testing strategies to enhance the accuracy of AST predictions and inform more effective treatments for *A. baumannii* infections.

In this article, we describe two protocols to assess the impact of culture medium on *A. baumannii* AST results and biofilm formation. Basic Protocol 1 details how to compare MIC values obtained via the traditional gold‐standard BMD methods following exposure of *A. baumannii* isolates to active agents in bacteriological (MHB) versus physiological (RPMI) media. Basic Protocol 2 outlines how to assess biofilm formation under host‐mimicking conditions by comparing the biofilm‐forming capacity of *A. baumannii* isolates in RPMI, tryptic soy broth (TSB), and MHB, with and without antimicrobial exposure, using a crystal violet assay

## Basic Protocol 1 COMPARING *A. baumannii* MINIMUM INHIBITORY CONCENTRATION (MIC) RESULTS IN BACTERIOLOGICAL (MHB) VERSUS PHYSIOLOGICAL (RPMI) MEDIA

**Figure 1:**
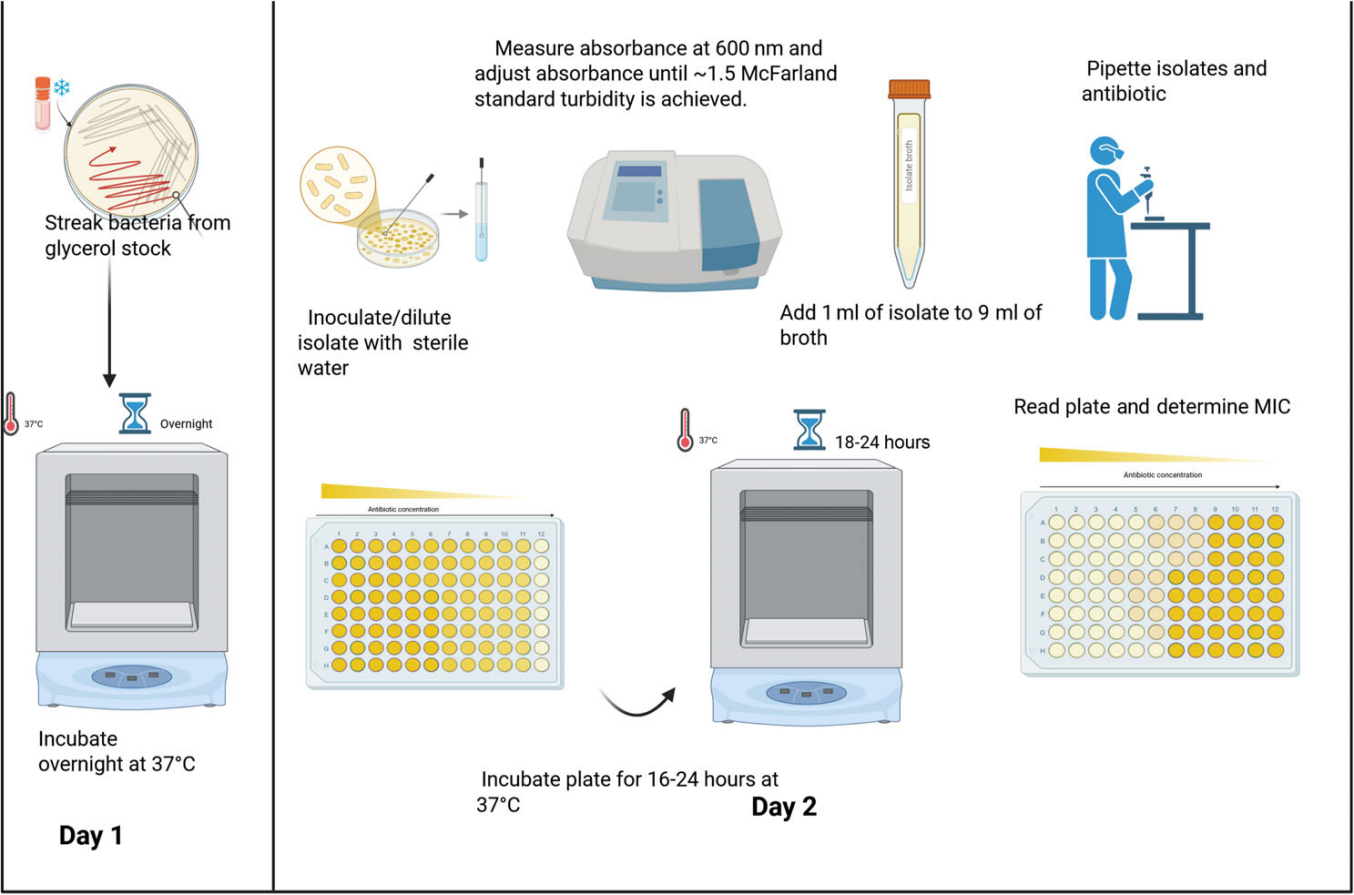
Workflow for comparing *A. baumannii* MIC results in MHB vs. RPMI using the BMD method. Overview of the 2‐day workflow to complete MIC testing for *A. baumannii* isolates in bacteriological (TSB and MHB) and physiological (RPMI) media. On day 1, *A. baumannii* isolates are streaked from cryogenic (glycerol) stock onto agar plates and incubated overnight at 37°C. On day 2, colonies are suspended in sterile water, and the turbidity is adjusted to ∼1.5 McFarland standard by diluting with sterile water. A 1:10 dilution of the suspension is then prepared and added to microtiter wells containing serially diluted antimicrobials in either MHB or RPMI. Plates are incubated for 16‐24 hr, and MICs are determined based on visible inhibition of growth. Based on CLSI guidelines.

Basic Protocol 1 describes how to perform MIC testing with *A. baumannii* using active antimicrobial agents and the gold‐standard BMD method for AST. The protocol also outlines how to interpret and present MIC results obtained from testing in bacteriologic culture medium (Mueller Hinton Broth, MHB) and physiological culture medium (RPMI 1640); the workflow is outlined in Figure 1. To perform MIC testing via BMD, clinical isolates should be recovered from cryogenic stocks, diluted in sterile water, adjusted to a turbidity of 1.5 McFarland units (∼1.5 × 10^8^ CFU/ml), and diluted 1:10. The isolates are then exposed to serially diluted *A. baumannii‐*active antimicrobials in both MHB and RPMI and incubated overnight at 37°C. MIC values from each medium should be recorded and analyzed to determine how culture conditions influence AST results. Although this protocol was applied specifically to evaluate MIC differences in 15 multidrug‐resistant (MDR) *A. baumannii* isolates collected during an outbreak at Loma Linda University Medical Center (LLUMC), it is readily adaptable to other Gram‐negative pathogens and antimicrobial agents. In this study, colistin (COL), a commonly used last‐resort agent for *A. baumannii*, was used as the test antimicrobial. Therefore, COL will be referenced throughout to illustrate the interpretation and presentation of results.

### Materials

Cryogenic stocks of *Acinetobacter baumannii* isolate(s) of interest
McFarland Standard Kit (ThermoFisher, cat. no. R20241)
Tryptic soy agar (see recipe) or other nutrient‐rich agar plates (prepared in 100 × 15‐mm petri dishes, Fisher, cat. no. FB0F875712)
Sterile water (ultrapure; obtained using the Barnstad Nanopure filtration system, Thermo Fisher, cat. no. D11951)
0.9% (w/v) saline (see recipe)
Roswell Park Memorial Institute medium (RPMI 1640; Gibco, cat. no. 11875119)
Mueller Hinton II Broth (MHB; BD, cat. no. 212322; see recipe)
Antimicrobial agent(s): e.g., colistin (Fisher Scientific, cat. no. AAJ6669803) and ampicillin‐sulbactam (ampicillin, Sigma Aldrich, cat. no. S9701; sulbactam, Sigma Aldrich, cat. no. 1033000)
Quality control strains (American Type Culture Collection [ATCC])
Laminar flow hood (BSC Class II, or equivalent)
Inoculating loops, sterile (Fisher, cat. no. 22363600)
Incubator (Binder BD)
15‐ml conical tubes
Disposable cuvettes
Disposable droppers
UV‐visible spectrophotometer (Mettler‐Toledo)
96‐well round‐bottom microtiter plates, non‐treated (GenClone, cat. no. 25‐224, or Corning, cat. no. 351177)
50‐, 200‐, and 1000‐µl single‐channel pipets (Brand Transferpette)
50‐µl multichannel pipets (Transferpette)
Plate reader (Bio‐Rad Xmark Spectrophotometer)

#### Day 1 prep: Culture bacteria on agar plates

1 Streak bacteria isolate of interest from cryogenic stock onto a nutrient‐rich agar plate using sterile inoculating tips.
  We use tryptic soy agar, but any general agar medium can be used.
  Ensure plates are accurately labeled.
  Proper technique is needed to obtain singular isolated colonies.
2 Incubate plates at 35°C ± 2°C for 16‐20 hr (or until colonies form).
  For non‐fastidious bacteria, cultures may be stored at 4°C for up to 48 hr at this point.

#### Day 2: Perform MIC testing using the BMD method in both RPMI and MHB media

Refer to CLSI guidelines (Lewis et al., 2025) for MIC breakpoints, quality control strains, etc.

**Figure 2:**
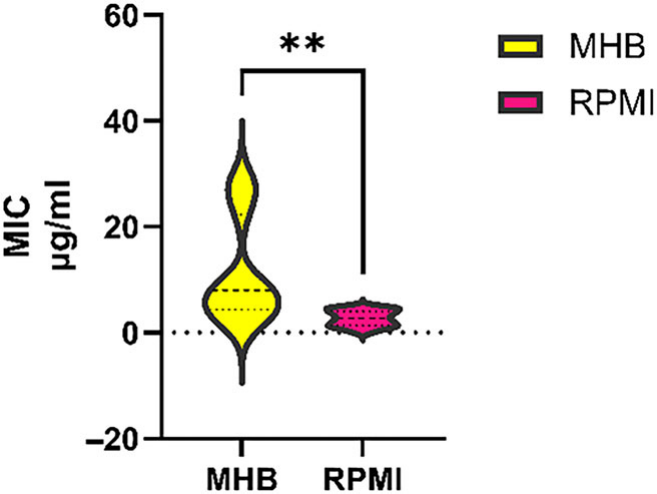
Impact of culture medium on MIC results for colistin (COL) against MDR *A. baumannii*. Graphical comparison of the MIC values obtained for COL against 15 MDR *A. baumannii* isolates in bacteriological medium (MHB; yellow) and physiological medium (RPMI; magenta). Violin plots illustrate the distribution of MIC values across the two medium conditions. A paired *t*‐test was used to assess the statistical significance of medium‐dependent differences in MIC results. Statistical significance was defined as *p* < .05 (indicated by **).

3 Label tubes.
4 Add 3‐5 ml sterile water or 0.9% saline to labeled conical tubes.
5 Suspend bacteria by streaking a lawn of colonies from cultured bacteria using an inoculating loop.
6 Measure and adjust absorbance at 600 nm to ∼1.5 McFarland units. Record absorbance values.
7 Dilute bacterial suspension in medium (setting up separate dilutions in MHB and RPMI) at a 1:10 ratio of bacteria to medium. These are your bacterial (bug) stocks. Label bug stocks.
8 Make drug stock: Constitute antibiotic to a concentration of 1 mg/ml in sterile water or 0.9% saline.
9 Determine the amounts of media and antibiotic needed using the following:
  Amount of active agent (AA) = 8∗A∗V
*D* = *V* – AA
  where *A* is the highest desired antibiotic concentration, *V* is the total final volume, AA is the amount of active agent, and *D* is the volume of medium to which AA will be added.
10 Add AA of drug stock to *D* of medium (DM). Label as drug in medium (DM).
11 Label and map 96‐well round‐bottom microtiter plate(s).
  Ensure that you have growth control wells, blank well(s), and CLSI sterility control wells
12 Add 50 µl medium to each well across the microtiter plate.
13 Add 50 µl DM into the highest‐concentration column of wells.
*Ideally drug concentrations should decrease* *from left to* *right*.
14 Serially dilute by a factor of 2 across the plate.
  Remember that NO DRUG goes into the growth control column of wells.
  Remember that NO BUG or DRUG goes into the sterility control column of wells.
15 Add 50 µl bug stock to each well.
16 Ensure that final volume in each well is 100 µl (based on CLSI guidelines; Lewis et al., 2025).
17 Incubate microtiter plate at 37°C for 16‐24 hr.
  Plates with RPMI should be incubated in 5% CO_2_ to maintain physiological pH.
18 Determine MIC by identifying the first well without visible bacterial growth (turbidity) or lacking a “pellet” at the base of the medium.
19 Record MIC.
20 Compare MICs generated in the different media, as shown in Figure 2.
  For “population” statistics, use unpaired Student's t‐test, with a p‐value cutoff of p = .05 for assigning significance.
  For isolate‐specific comparisons, use paired Student's t‐test, with a p‐value cutoff of p = .05.

## Basic Protocol 2 COMPARING *A. baumannii* ISOLATE BIOFILM FORMATION FOLLOWING ASSAYS COMPLETED IN BACTERIOLOGICAL CULTURE MEDIA (MHB AND CONTROL TSB AND PHYSIOLOGICAL MEDIUM (RPMI)

**Figure 3:**
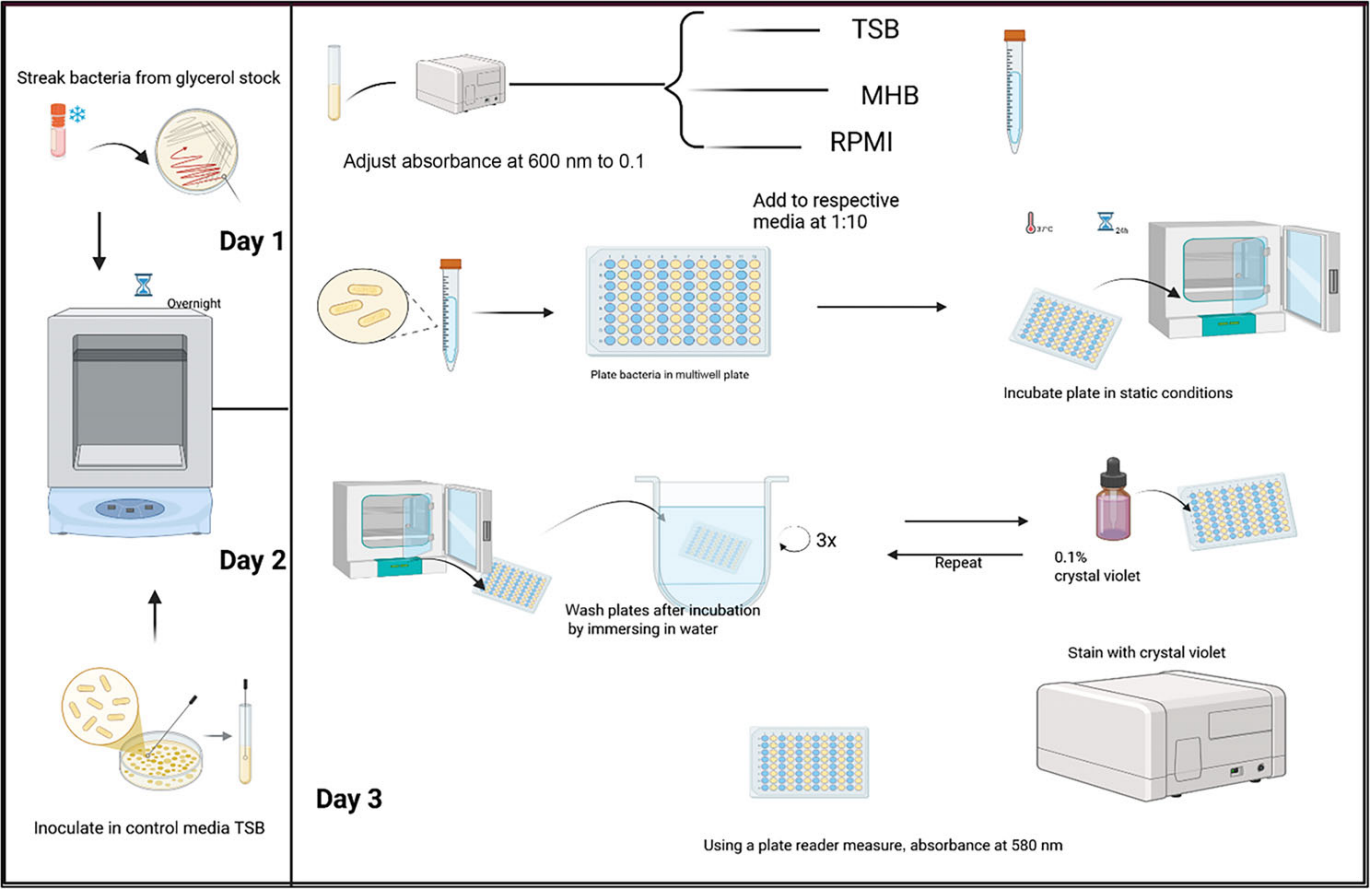
Workflow for comparing *A. baumannii* biofilm formation in different culture media. Overview of the 3‐day experimental workflow for assessing biofilm formation in bacteriological (TSB and MHB) and physiological (RPMI) media. On day 1, *A. baumannii* clinical isolates are streaked from glycerol stocks onto agar plates and incubated overnight at 37°C. On day 2, isolate(s) are inoculated into TSB and incubated overnight at 37°C. On day 3, cultures are adjusted to an absorbance at 600 nm of 0.1 via dilution with sterile water; further diluted 1:10 in TSB, MHB, or RPMI, with or without antimicrobial and active agent or non‐treated; and plated in 96‐well microtiter plates. Plates are incubated for 24 hr under static conditions. Plate wells are then gently washed by immersion in distilled water, stained with 0.1% crystal violet, rinsed, dried, and then destained with ethanol. Biofilm formation is quantified by measuring absorbance at 580 nm using a plate reader.

Basic Protocol 2 describes how to assess the biofilm‐forming capacities of *A. baumannii* isolates in bacteriological (MHB and TSB) and physiological (RPMI) culture media, both in the presence and absence of active antimicrobial agents (for workflow, see Fig. 3). The protocol also outlines how to interpret and present the resulting data. To begin, overnight cultures of *A. baumannii* should be transferred into different bacteriological media, such as TSB, MHB, and RPMI, with TSB serving as a control to account for potential confounding effects of overnight growth conditions. The cultures are tested with and without *A. baumannii‐*active antimicrobials. After overnight incubation, bacterial suspensions are plated into wells of 96‐well microtiter plates and incubated statically at 37°C for 24 hr. Wells are then washed with distilled water, stained with 0.1% crystal violet (CV), rinsed, dried, and destained with ethanol. Biofilm biomass is quantified by measuring absorbance. This protocol was initially developed to assess biofilm formation in 15 multidrug‐resistant *A. baumannii* isolates. However, it is readily adaptable for evaluating biofilm dynamics in other Gram‐negative organisms. In our experiments, colistin (COL) was used as the representative antimicrobial; thus, COL is referenced throughout to illustrate how to present and interpret results.

### Materials

Cryogenic stocks of *A. baumannii* isolate(s) of interest
Tryptic soy broth (TSB; see recipe)
Mueller Hinton Broth (MHB; see recipe)
Roswell Park Memorial Institute (RPMI 1640; Gibco, cat. no. 11875119, or GenClone, cat. no. 25‐506)
Tryptic soy agar (TSA; see recipe)
Antimicrobial(s) active against *A. baumannii* (e.g., colistin)
Sterile water (ultrapure; prepared using the Barnstad Nanopure filtration system from Thermo Fisher, cat. no. D11951)
0.9% (w/v) saline (see recipe)
Chlorine solution/bleach
70% (v/v) ethanol (see recipe)
Crystal violet (see recipe)

Laminar flow hood (Labconco Purifier BSC Class II or equivalent)
Sterile inoculating loops (Fisher, cat. no. 22363600)
Incubator (Binder RD)
15‐ml conical tubes (Greiner Bio‐one)
Disposable cuvettes
UV‐visible spectrophotometer (Mettler‐Toledo)
200‐ and 1000‐µl single‐channel pipets
Permanent marker
96‐well flat‐bottom microtiter plates, non‐treated (GenClone, cat. no. 25‐104, or Corning, cat. no. 351172)
50‐µl multichannel pipets
Waste beaker
Plate reader: Bio‐Rad Xmark Spectrophotometer

#### Day 1: Culture bacteria on agar plates

1 Streak bacteria isolate of interest from cryogenic stock onto a nutrient‐rich agar plate.
  We use tryptic soy agar, but any general/basal agar medium may be used.
  Ensure plates are accurately labeled.
  Proper technique is needed to obtain singular isolated colonies.
2 Incubate plates at 37°C for 16‐20 hr or until colonies are seen
  For non‐fastidious bacteria, cultures may be stored at 4°C for up to 48 hr at this point.

#### Day 2: Inoculate isolate(s) in tryptic soy broth TSB

3 Label appropriate tubes.
  We use 15‐ml centrifuge tubes. Make sure to include a negative control (NC).
4 Add 3‐5 ml of TSB into tube.
5 Using a sterile loop, inoculate 3‐5 colonies of the bacterial isolate of interest in TSB.
6 Place in incubator overnight.

#### Day 3: Experimental setup and treatment

7 Label six sets of tubes (1 set = number of bacterial isolates + 1 NC). There should be a “no‐treatment” control group (NT) and treatment group (AA) for each type of medium (TSB, MHB, and RPMI).
8 *Optional*: Perform sub‐inhibitory concentration exposure.
  a. Using MHB MICs, determine 40^th^‐percentile MIC.
  b. Identify classification based of breakpoint value (according to CLSI guidelines; Lewis et al., 2025).
  c. Determine final desired concentration (FDC) by dividing breakpoint value by 2.
9 Determine the amount of medium needed in treatment tubes.
$$\mathrm{MedT}=V-\;\left(\frac{\mathrm{FDC}\;*V}{\mathrm{Abx}}+0.1\left(V\right)\right)$$
  where MedT is the amount of medium with the treatment, MedNT is the amount of medium without treatment, FDC is the final desired concentration (µg/ml), *V* is the total volume, and Abx is the drug stock concentration.

For combination therapies, use 0.5 FDC for each antimicrobial treatment.

10 Add MedT of medium to the respective tubes.
  MedT is the same for all three media.
11 Add MedNT (0.9 *V*) of medium to the respective tubes.
12 Retrieve overnight isolates.
13 Measure absorbance at 600 nm and dilute bacterial suspension to adjust the absorbance to ∼0.10.
14 Dilute bacterial suspension in medium in 15‐ml conical tubes at a 1:10 ratio, where 10 = *V*.
*To* *all* *tubes (both MedT and MedNT)*.
15 Make drug stock:
  a. Constitute antimicrobial to desired concentration in sterile water or 0.9% saline to make the drug stock (0.2 mg/ml for all active agents except ampicillin/sulbactam (AS) if applicable, for which it should be 0.4 the con == mg/ml).
  b. Add drug (DM) to treatment (MedT) tubes.
$$\mathrm{DM}\;=\;\frac{\mathrm{FDC}*V}{\mathrm{Abx}}$$
16 Label and map flat‐bottomed 96‐well microtiter plates.
  Edge wells should not be utilized in this experiment, to avoid the edge effect resulting in unreliable results. Ensure all controls are present: sterile control (medium), negative control (medium with antimicrobial), and positive control (reference strain without antimicrobial).
17 Transfer 150 µl of bacterial culture (treatment and NT) into each plate well according to the plate map.
18 Record time after the setup of each plate is complete.
19 Incubate plates at 37°C for 24 hr.
  When testing in RPMI, incubate at 37°C, 5% CO_2_ to maintain physiological pH.

#### Biofilm staining

20 Measure plate absorbance at 600 nm (*A*_600_; this informs on growth).
21 Add bleach to waste beaker.
22 Dump the contents of the microtiter plates into waste beaker.
23 Wash plates by gently immersing in sterile water, preferably deionized (DI) water.
24 Repeat steps 22 and 23 three times.
25 Place microtiter plate face down on drying mat, paper towels, or similar. Gently tap the bottom of the plate and leave to dry.
26 Once dry, add 175 µl of 0.1% (w/v) crystal violet to each well.
27 Stain for at least 35 min.
28 Repeat steps 20‐25.
29 Once plate is dry, add 200 µl of 70% (v/v) ethanol to wells.
  This dissolves crystal violet stained biofilm.
30 Leave for at least 35 min.
31 Add 100 µl of 70% ethanol to wells in row H.
32 Add 100 µl of 0.1% crystal violet to well H1.
33 Serially dilute the contents of well H1 across row H.
34 Top up wells to 200 µl with appropriate medium.
  This is the crystal violet standard, which allows you to check for linearity of your results.

#### Data collection, processing, and analysis

**Figure 4:**
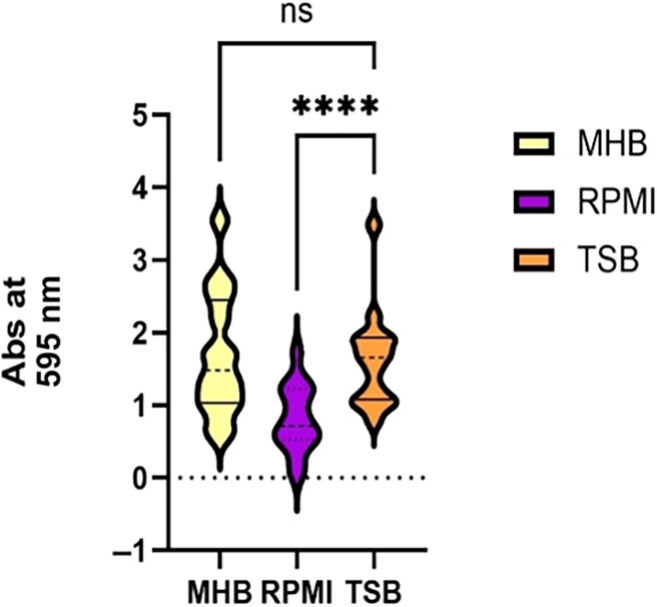
Impact of culture medium on *A. baumannii* biofilm formation in the absence of antimicrobial exposure. Graphical comparison of biofilm biomass for 15 multidrug‐resistant *A. baumannii* isolates cultured in bacteriological (MHB, TSB) and physiological (RPMI) media, without exposure to the active agent colistin (COL). Biofilm formation was assessed using a crystal violet assay, and absorbance was measured at 595 nm. Violin plots represent the distribution of absorbance values across conditions. To evaluate the effect of medium on biofilm biomass, mean absorbance values were compared using two‐way ANOVA. Statistical significance was defined using a *p*‐value cutoff of < .05. (**** indicates *p* < .05).

35 Using plate reader, measure absorbance at 595 nm.
  If plate reader has a “mix” step that combines the contents in the well into a homogenous mixture before the reading, mix for at least 10 sec.
36 Record absorbance values for each isolate in each medium.
37 To assess the impact of medium alone on biofilm formation, use the no‐treatment (NT) data results.
  A p < .05 denotes statistical significance, as shown in Figure 4.

**Figure 5:**
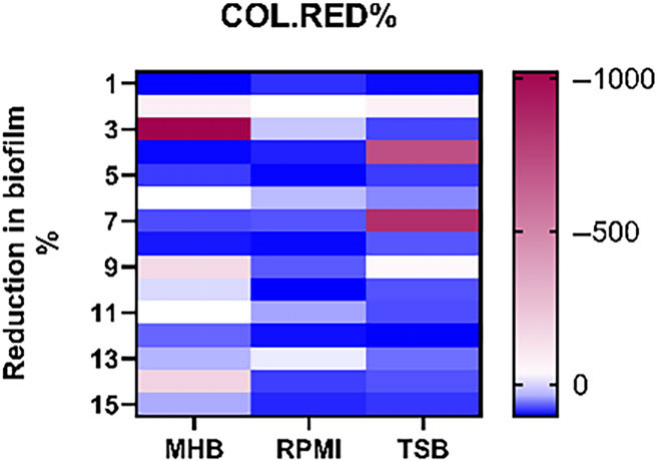
Impact of culture medium on *A. baumannii* biofilm formation following COL exposure. Graphical comparison of biofilm formation by 15 multidrug‐resistant *A. baumannii* isolates cultured in bacteriologic (MHB) and physiologic (RPMI) media following exposure to active agent, colistin (COL), here used as a reference antimicrobial. Biofilm biomass was quantified using crystal violet staining, and the percentage reduction in biofilm formation relative to untreated controls was calculated to assess the antimicrobial effects across different media. Differences were evaluated using two‐way ANOVA, and a heatmap can be used to visualize isolate‐specific responses and highlight the influence of media and/or antimicrobial treatment. Statistical significance was defined using a *p*‐value cutoff of < .05. (**** indicates *p* < .05).

38 To assess the impact of the antimicrobial alone on biofilm formation, compare the data from the no‐treatment (NT) and treatment (T) assessments completed in the same medium (either TSB, MHB, or RPMI).
  Calculating the percentage reduction within the medium (either TSB, MHB, or RPMI) provides this information. The equation to utilize for this calculation is the following, where CV = crystal violet absorbance.
$$\mathrm{Percentage}\;\mathrm{reduction}\;=\;\frac{\mathrm{CVnt}\;-\;\mathrm{CVt}}{\mathrm{CVnt}}*100$$
  A p < .05 denotes statistical significance, and results can be visualized using a heat map as shown in Figure 5.

## REAGENTS AND SOLUTIONS

### Saline, 0.9% (w/v)

Add 9 g sodium chloride (Sigma‐Aldrich, 746398) to a final volume of 1 L of water. Autoclave for 30 min at 121°C. Store up to 3 months after opening at room temperature.

### Crystal violet, 0.1% (w/v)

Dissolve 1 g of crystal violet powder (Fisher Scientific, cat. no. C581‐100) in 200 ml of ethanol (Lab Alley 200 proof). Shake vigorously or vortex for ∼15 s. Add sterile water (DI) to a final volume of 1 L (20% ethanol [v/v]). Store up to 1 year in a cool, dark place, preferably in an amber bottle.

Always validate performance if solution is older than 3 months

### Ethanol, 70% (v/v)

Add 700 ml 100% ethanol (Lab Alley, 200 proof) to 300 ml sterile water (DI) in a 1‐L bottle. Store indefinitely in a cool, dry place.

### Mueller Hinton II Broth (MHB)

Suspend 22 g of the powder (BD DIFCO, cat. no. 212322) in 1 L of purified water, mix thoroughly, heat with frequent agitation, and boil for 1 min to completely dissolve the powder. Autoclave at 116°C‐121°C for 10 min.

This should be done before day 2.

### Tryptic Soy Agar (TSA)

Suspend 40 g TSA powder (BD DIFCO, cat. no. 236950) in 1 L of purified water; mix thoroughly. Boil to completely dissolve the powder. Autoclave at 121°C for 15 min. Store indefinitely at −20°C.

### Tryptic Soy Broth (TSB)

Suspend 30 g TSB powder (BD DIFCO, cat. no. 211822) in 1 L of purified water; mix thoroughly. Warm slightly to completely dissolve the powder. Autoclave at 121°C for 15 min. Store up to 3 months after opening at room temperature. Do not use if there are signs of contamination.

## Commentary

### Critical Parameters

The current gold standard for antimicrobial susceptibility testing (AST) includes determining minimal inhibitory concentrations (MICs) using the broth microdilution (BMD) method in bacteriological medium (MHB), according to CLSI guidelines (Lewis, 2025). All experiments described in Basic Protocol 1 should follow these guidelines, with the only deviation being the substitution of MHB with physiological media (RPMI) in parallel testing.

To ensure high testing precision and accuracy, testing should occur in non‐tissue‐culture‐treated (non‐treated) plates, as tissue‐culture‐treated plates may aid antibiotic adsorption onto the plates, affecting results. Additionally, when testing in RPMI media, incubation should be performed in 5% CO_2_ to maintain physiological pH.

The MIC values obtained are used to determine sub‐inhibitory concentrations for subsequent biofilm assays in Basic Protocol 2, making it essential to perform MIC testing before biofilm assessment. *A. baumannii* MIC testing via BMD has an approximate turnaround time of 24 hr. To ensure a consistent inoculum, clinical isolates should be cultured from cryogenic stock, and suspensions should be adjusted within the doubling time of the organism. The doubling time (DT) can be calculated using the formula:

$$\mathrm{DT}=\frac{\left(T_{2\;-}\;T_{1}\right)*\ln\left(2\right)}{\ln\left(\mathrm{CF}U_{2}-\mathrm{CF}U_{1}\right)}$$

In our study, the MDR *A. baumannii* isolates had an average doubling time of ∼50 min; thus, suspensions were seeded within ∼40 min of preparation (Antunes et al., 2011). Antimicrobial stock solutions should be prepared fresh daily to ensure consistency in final concentrations. Variability in stock preparation can affect reproducibility and interpretation of results.

During biofilm assays (Basic Protocol 2), include negative controls during day 2 overnight inoculations for quality control and contamination monitoring. Negative controls may contain drug, while sterile/blank wells should contain only medium. These should be clearly distinguished during data analysis. Handle microtiter plates gently to avoid biofilm disruption and minimize assay variance. Overstaining with CV can increase biofilm fixation but lead to uneven quantification; similarly, ensure that the destaining step is fully complete before measuring absorbance to achieve homogenous readings. Lastly, avoid using the outer wells of the 96‐well microtiter plates in testing for biofilm formation, as they are prone to evaporation, which can affect results.

### Troubleshooting

For troubleshooting suggestions, refer to Table 1.

**Table 1:** Troubleshooting Guide for Enhancing Antimicrobial Susceptibility Testing for *A. baumannii* isolate(s) Using Physiologically Relevant Culture Media and Biofilm Formation Assays

**Problem**	**Possible cause**	**Solution**
**Basic Protocol** **1**	(To be used in conjunction with CLSI M‐100)	
Precipitation in RPMI	Partial insolubility of antimicrobial	Dissolve drug in DMSO instead of sterile water/0.9% saline.
**Basic Protocol** **2**		
Sample *A*_600_ values equal sterility control	No bacteria added	Repeat experiment; verify inoculation step.
Growth in sterile wells	Contamination during plating	Repeat experiment; disinfect all working areas thoroughly.
Growth in negative control tubes after overnight incubation	Contamination during Day 2	Repeat day 2 overnight steps; ensure aseptic technique is followed.
Growth in negative control wells; NO prior growth in negative control tubes; ALL sterile wells are clear	Contamination during day 3, steps 11‐13, for NC	As ALL sterile wells are clear, there is no contamination; record contamination during day 3. Stain as usual. Adjust plate reader template to replace 1 sterile well with NC.
Overstained well	Spillover during experimental set up	Exclude data point; perform additional if needed.
Biofilm loss during washing	Disruption of biofilm	Reduce wash steps; use multi‐channel pipet instead of immersion.
Absorbance at 595 nm not in the linear region based in standard	Excessive CV staining	Dilute the solubilized CV in both treated and control groups by 50%.

### Understanding Results

To assess the impact of culture medium on *A. baumannii* MIC values, we followed the procedures described in Basic Protocol 1. We observed a significant difference in colistin susceptibility (as shown in Fig. 2). Conducting a paired *t*‐test is recommended to account for intra‐isolate variability and increase statistical power.

To assess the impact of culture medium on *A. baumannii* biofilm formation, we followed the procedures described in Basic Protocol 2. In our study, we observed a significant difference in biofilm formation across media. Conducting a two‐way ANOVA to evaluate the independent and interactive effects of culture medium and antimicrobial exposure on biofilm mass is recommended. Results can be visualized in GraphPad Prism using a violin plot (as shown in Fig. 4) or a heatmap (as shown in Fig. 5) to illustrate isolate‐specific responses.

To evaluate the effect of treatment, the percent difference in absorbance between treated (AA) and nontreated (NT) wells should be calculated. This normalizes for growth variation across media. Percent difference values should be compared using two‐way ANOVA. A significant main effect (*p* < .05) indicates an independent influence of either medium treatment. A significant interaction effect (*p* < .05) suggests an additive or synergistic impact of both variables.

### Time Considerations

This protocol enhances the physiological relevance of AST but does not reduce turnaround time. It provides a more comprehensive framework by integrating AST with biofilm dynamics.

- Culturing from cryogenic stocks: ∼20 hr
- MIC testing (setup + incubation + reading): ∼28‐30 hr total
- Biofilm assay (day 2 inoculation + 24 hr static incubation + crystal violet staining and analysis): ∼48 hr

Overall, MIC testing takes ∼48 hr, and the full biofilm protocol requires an additional 48 hr, resulting in a total time of ∼96 hr from initial culturing to final results.

### Author Contributions

**Nana Yaa P. Sakyi** Opoku: Conceptualization; data curation; formal analysis; investigation; methodology; validation; visualization; writing—original draft; writing—review and editing. **Arunima Mishra**: Data curation; methodology; validation; writing—review and editing. **Hansel Fletcher**: Conceptualization; formal analysis; funding acquisition; investigation; methodology; project administration; resources; supervision; Visualization; writing—original draft; writing—review and editing. **Victor Nizet**: Conceptualization; project administration; resources; Supervision; writing—review and editing. **Jacinda C. Abdul‐Mutakabbir**: Conceptualization; data curation; investigation; methodology; project administration; resources; supervision; writing—original draft; writing—review and editing.

### Conflict of Interest

J.A.M. has served on advisory boards and received honoraria from Shionogi, GSK, and Pfizer. V.N. has served on advisory boards and holds stock options in Cellics Therapeutics, I2 Pure, and Clarametyx Biosciences, and his intellectual property has been licensed via the University of California, San Diego to Vaxcyte, Inc.

## Data Availability

Data supporting the findings of this study are available from the corresponding author upon reasonable request.
